# Elevated band count in the pediatric patient

**DOI:** 10.3389/fped.2025.1483929

**Published:** 2025-05-09

**Authors:** Aaron Grubner, Jennifer E. Sanders, Regina M. Longley, Maria Vergara-Lluri

**Affiliations:** ^1^ Department of Emergency Medicine, Icahn School of Medicine at Mount Sinai, New York, NY, United States; ^2^ Department of Pediatrics, Icahn School of Medicine at Mount Sinai, New York, NY, United States; ^3^ Icahn School of Medicine at Mount Sinai, New York, NY, United States; ^4^ Department of Pathology, Keck School of Medicine, University of Southern California, Los Angeles, CA, United States

**Keywords:** bandemia, band count, left shift, pediatrics, immature neutrophils

## Abstract

**Introduction:**

In this review article we survey the literature for current evidence in pediatric practice regarding the use of elevated band count in the pediatric emergency room. In addition, we present data from the literature on the wide variability of manual band counts to reconsider its utility in clinical practice.

**Background:**

Bandemia is commonly seen during a state of infection. Band count is determined by manual cell count and can be prone to inaccuracy and imprecision. Despite its shortcomings, the 100-cell manual differential count remains the most practical method for assessing left shift.

**Methods:**

All the literature involving the use of elevated band count as a biomarker in pediatrics available on PubMed and Google Scholar was surveyed. “Bandemia”, “Band count”, “left shift” and “immature neutrophils” were used as primary search terms, in conjunction with the term “pediatrics.”

**Results:**

The most recent AAP guidelines do not incorporate band count in decision making for febrile neonates. Elevated band count is related to worse outcomes in non-operative management of appendicitis. Elevated band count can be seen in viral illness alone. Even severe bandemia (<20%) does not correlate with severe illness.

**Discussion:**

More studies are needed to definitively dispel the notion of bandemia and its association with invasive bacterial infection. Additionally, pediatric providers may benefit from professional society guidelines advising appropriate management of the pediatric patient with elevated band count.

## Introduction

Identifying patients with serious bacterial infections remains a challenging aspect of pediatric medical practice. There is debate about the utility of measuring band count in clinical practice (1). Nonetheless, during the 1991 American College of Chest Physicians/Society of Critical Care Medicine Consensus Conference, greater than 10% bands on a complete blood count (CBC) was included in the sepsis criteria (2). In 2002, a panel of international experts applied that criteria to pediatric patients (3). Newer pediatric sepsis guidelines don't include bandemia as a criterion for sepsis (4), however, many clinicians continue to consider an elevated band cell level as a surrogate for serious bacterial illness. Studies in adult populations confirm that >10% bands correlates with progression to septic shock and bacteremia (5, 6). Additional studies in adult populations have shown that the degree of bandemia, especially in the setting of concurrent tachycardia or fever, is associated with greater likelihood of 30-day mortality (7). However, studies in pediatric populations are not as robust. In this review article, we survey the literature for current evidence in pediatric practice regarding the use of elevated band count in the pediatric population. In addition, we present data from the literature on the wide variability of manual band counts to reconsider its utility in clinical practice.

## Background

### What are bands?

**Figure 1:**
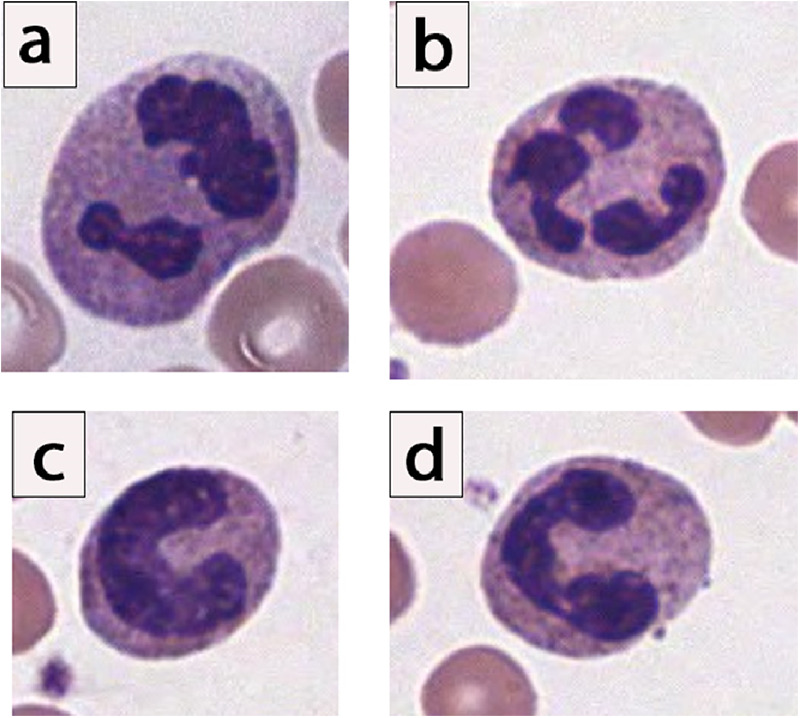
Morphology of neutrophils, showing spectrum of segmented and band neutrophils. **(a,b)** Examples of mature segmented neutrophils, given the clear multilobation and threadlike filaments lacking chromatin. **(c)** Example of an “easy” band neutrophil, given the C-shaped configuration of the nuclei, which is indented to more than half the distance to the farthest nuclear margin. **(d)** Example of a “moderately challenging” band neutrophil. Notice that there is constriction connecting 2 lobes, yet clearly visible chromatin is noted in between the dark, parallel nuclear margins. In surveyed laboratory responses, this “moderately challenging” neutrophil would have been called a “band” neutrophil by only 25% to 39% of laboratories (9).

Neutrophils are one of the body's five circulating white blood cells. The mature neutrophil contains a segmented nucleus, typically with two to five lobes (8). Immediately prior to the mature, segmented form, the neutrophil is in its “band” form, and its nucleus lacks segmentation (Figure 1).

A small number of band cells can be seen in normal blood circulation, however reference ranges are controversial. Band counts are highest in the neonatal period, with some authors listing up to 18% bands as a neonatal physiologic norm (1). Band cells then diminish rapidly in the first two weeks after birth. Because of the rapid fluctuation in band count during the neonatal period, band count is seldom used clinically during the neonatal period (1). Thereafter, the band count slowly drops and will resemble adult levels by approximately five years of age (1). Reference ranges reported in older children and adults may vary, with authors using anywhere from 4% up to 11% as the normal range (10, 11). Regardless, in states where the body is hastening to release neutrophils to the periphery, a higher percentage of band cells may be seen on a peripheral blood sample (12).

Bandemia is commonly seen during a state of infection, and, interestingly, band cells have been shown to have superior antibacterial capacity *in vitro* (13). This has led to a hypothesis that banded neutrophils are not released as bystanders, but are deliberately released as cells highly adept at pathogen killing (13). Some of the earlier forms of immature neutrophils such as metamyelocytes, and rarely myelocytes, may also be seen during infections (14) and leukemoid reactions. Forms less mature than the myelocyte (e.g., promyelocytes, myeloblasts) are suspicious for malignancy and may warrant a bone marrow biopsy (14, 15). However, bandemia is also reported in malignancies (acute leukemia, myeloproliferative neoplasms) and reactive non-infectious inflammatory states such as stress responses, and tissue damage and necrosis (1).

#### Band cell terminology

Some of the terminology surrounding immature neutrophils can, at times, be a source of confusion so it is worthwhile to elaborate. The term *left shift* is somewhat ill-defined, but typically refers to an increase or shift towards any of the immature neutrophil forms. There are competing explanations for the origin of the phrase *left shift,* including the left-most button arrangement of early cell sorting machines and a 1920s publication by Josef Arneth, containing a graph in which immature neutrophils with fewer segments shifted the median left (16). The term *left shift* should be distinguished from *neutrophil predominance* and *neutrophilia*, terms which do not refer specifically to immature cells, but rather refer to a state where a large proportion of the white blood cells are made up of neutrophils (12). Finally, the term *immature granulocyte percentage* (IG%) is reported on some automated analyzers as part of a six-part CBC differential and refers to immature forms of all granulocytes (i.e., promyelocyte, myelocyte, and metamyelocyte precursors of neutrophils, eosinophils and basophils). However, it should be noted that neutrophils typically account for greater than 96% of all granulocytes (12).

#### Technical limitations of the manual measurement of bands

Automated hematology analyzers offer automated, accurate, and precise differential counts of the five to six subclasses of leukocytes, with thousands of WBCs counted by the analyzer to generate counts (17). If the number of immature cells are high enough to trigger the detection threshold of the analyzer (i.e., an elevated *immature granulocyte percentage*), the automated analyzer will flag, and the technician will be alerted. However, the analyzer is unable to distinguish specific cells in the developmental sequence of granulocytes and are therefore unable to provide a band count (18). When the automated analyzer flags a specimen, a manual microscopic review is needed.

Manual cell counting by the clinical laboratory is a time-consuming, labor-intensive process involving slide preparation followed by a 100-cell manual leukocyte tabulation by a trained laboratory technologist or scientist. Some problems associated with the manual band count include: the inherent statistical imprecision of a 100-cell count (9), dissimilar definitions of bands (19), and variation owing to nonhomogeneous distribution of leukocytes on the blood film (20). Furthermore, the interpretation is technician dependent, and its enumeration can be associated with inaccuracy and imprecision, even when operators are trained on a single definition (21–23). The subjective nature of this examination procedure has led some to conclude that even if the same person were to examine a given blood ﬁlm a second time, the percentage of each type of leukocyte would not invariably be the same (20).

A 2020 survey of more than 1,300 laboratories accredited by the College of American Pathologists (CAP) reaffirms the significant variability in the enumeration of the manual band counts (9). Although most participants could identify “easy” band neutrophils fairly well (mature neutrophils with no appreciable lobulation or segmentation), cell identifications for “moderate” and “difficult” bands were poor. Studies on the inherent fallibility of the manual band count, published predominantly in the pathology and laboratory medicine literature, have led to strong recommendations by the CAP and the Clinical and Laboratory Standards Institute to no longer enumerate band cells independently, and to count segmented and band neutrophils together (9, 24). Despite these many technical shortcomings, the 100-cell manual band differential count continues to be used as a surrogate for assessing left shift.

## Methods

A survey of the literature was completed by querying PubMed and Google Scholar for texts that address the use of elevated band count as a biomarker in pediatric patients. Specifically, “bandemia”, “band count”, “left shift” and “immature neutrophils” were used as primary search terms, in conjunction with the term “pediatrics.” If an article utilized these terms in the title and/or abstract, then the full text was screened. An article was included and data was extracted if there was a significant contribution to current clinical practice. Articles that discussed bandemia in neonates or adults exclusively were excluded.

## Results

### Bandemia in the pediatric patient: a survey of the literature

Clinically, an increase in band cells indicates that the body is mounting an appropriate inflammatory response to an infectious or inflammatory process. However, in the pediatric population, the relationship between an elevated band count and serious bacterial illness is unclear.

#### Neonates

The clinical use of band count in neonates with fever is of particular interest due to the concern for neonatal sepsis. Developing an evidence-based approach to the evaluation and management of febrile infants has been an ever-changing field, spanning more than four decades (25). Previous guidelines have, indeed, incorporated band count to identify high-risk infants (26, 27). However, in light of more recent studies, advances in testing, and changing bacteriology, the most recent AAP guidelines do not incorporate band count (28), and it subsequently does not play a large role in decision making for febrile neonates.

#### Infants and toddlers

In 1999, Kupperman et al. examined a cohort of 100 febrile children aged two years or younger with either laboratory-documented bacterial infections or laboratory-documented respiratory viral infections. When comparing these two groups, there was no difference in band count (29). The study concluded that the band count in the peripheral blood smear does not routinely help to distinguish bacterial infections from respiratory viral infections in young febrile children.

Isaacman et al. examined 633 pediatric patients aged three to 36 months of age who presented to the pediatric ER with fever. Forty-six of those patients were found to have positive blood cultures. When comparing CBC from the two groups, univariate analyses identified band count as significantly associated with an outcome of bacteremia, however this was not found to be significant in the final multivariate models (30).

#### Bandemia with appendicitis

Nonoperative therapy has been gaining popularity as an initial therapy in children with appendicitis. However, some studies have reported high failure rates when nonoperative therapy is chosen (31). In 2016, Talishinskiy et al. examined the factors leading to treatment failures and concluded that patients with an elevated band count were more likely to fail nonoperative therapy for appendicitis (32).

#### Severe bandemia

A band count of 20% or greater may be referred to as severe bandemia. Although in the adult population a bandemia of 20% or more was found to have five times significantly greater mortality (7), in the pediatric population this does not seem to be the case. In a study of 102 pediatric patients (two months to 18 years) with bandemia of greater than 20%, zero patients died, only one patient had a positive blood culture, and none had meningitis. The most common diagnosis was pneumonia (ten patients), and four patients had a UTI (33).

#### Bandemia with respiratory viruses

In the pediatric patient, an elevated band count can be seen solely from a viral respiratory illness. A study by Noyola et al. analyzed a cohort of 419 patients (of ≥one month and ≤five years) with confirmed respiratory viral infections and no other concomitant bacterial infection. Among that cohort, bandemia of >10% was found in about a quarter of cases of respiratory viral infections in the absence of concomitant bacterial infection, suggesting that viral illness alone can cause elevated band count. In addition, when comparing the band count between the viral group and a cohort of patients with confirmed bacterial illness, there was no statistically significant difference noted (34).

## Discussion

Over thirty years have passed since the Society of Critical Care Medicine Consensus Conference, which included a lab value of greater than 10% bands on a complete blood count as part of the sepsis criteria. This remains a commonly used value in clinical practice and a commonly included factor in clinical decision calculators for detection of pediatric sepsis (35). Pediatric physicians report band count as one of the most utilized laboratory values in recognition of sepsis (36), and, to this day, patients with a known viral infection, but high band count, are more likely to receive antibiotics and have higher hospital admission rates (34). These trends are seen despite the fact that numerous studies in the pediatric population seem to show that elevated band counts are not associated with bacterial illness. Pathology and laboratory medicine literature also demonstrates the low reliability of band counting, and leading pathology and laboratory medicine expert committees recommend discontinuation of separate band neutrophil reporting (9). The only study demonstrating significance of an elevated band count is related to non-operative management of appendicitis. In 2012, a pediatric patient with an unrecognized elevated band count died from sepsis, prompting regulations that require hospitals to adopt practices for the early identification and treatment of sepsis (37). The widespread awareness of the details of this story have potentially added to the discomfort and uneasiness of providers caring for a pediatric patient with an elevated band count. More studies are needed to definitively dispel the notion of bandemia and its association with invasive bacterial infection. Additionally, pediatric providers may benefit from professional society guidelines advising appropriate management of the pediatric patient with elevated band count.
